#  Combined Flow Cytometric Analysis of Surface and Intracellular Antigens Reveals Surface Molecule Markers of Human Neuropoiesis

**DOI:** 10.1371/journal.pone.0068519

**Published:** 2013-06-24

**Authors:** Gizem Turaç, Christopher J. Hindley, Ria Thomas, Jason A. Davis, Michela Deleidi, Thomas Gasser, Erdal Karaöz, Jan Pruszak

**Affiliations:** 1 Emmy Noether-Group for Stem Cell Biology, Department of Molecular Embryology, Institute of Anatomy and Cell Biology, University of Freiburg, Freiburg, Germany; 2 Center for Stem Cell and Gene Therapies Research and Practice, Kocaeli University, Kocaeli, Turkey; 3 Spemann Graduate School of Biology and Medicine (SGBM), Faculty of Biology, University of Freiburg, Freiburg, Germany; 4 Hertie, Institute for Clinical Brain Research, Department of Neurodegenerative Diseases, German Center for Neurodegenerative Diseases (DZNE), University of Tübingen, Tübingen, Germany; 5 Center for Biological Signaling Studies (BIOSS), University of Freiburg, Freiburg, Germany; National Institutes of Health, United States of America

## Abstract

Surface molecule profiles undergo dynamic changes in physiology and pathology, serve as markers of cellular state and phenotype and can be exploited for cell selection strategies and diagnostics. The isolation of well-defined cell subsets is needed for *in vivo* and *in vitro* applications in stem cell biology. In this technical report, we present an approach for defining a subset of interest in a mixed cell population by flow cytometric detection of intracellular antigens. We have developed a fully validated protocol that enables the co-detection of cluster of differentiation (CD) surface antigens on fixed, permeabilized neural cell populations defined by intracellular staining. Determining the degree of co-expression of surface marker candidates with intracellular target population markers (nestin, MAP2, doublecortin, TUJ1) on neuroblastoma cell lines (SH-SY5Y, BE(2)-M17) yielded a combinatorial CD49f^-^/CD200^high^ surface marker panel. Its application in fluorescence-activated cell sorting (FACS) generated enriched neuronal cultures from differentiated cell suspensions derived from human induced pluripotent stem cells. Our data underlines the feasibility of using the described co-labeling protocol and co-expression analysis for quantitative assays in mammalian neurobiology and for screening approaches to identify much needed surface markers in stem cell biology.

## Introduction

Flow cytometry offers a range of analytical and cell enrichment opportunities for basic and biomedical research and clinical applications [1]. Its utility is best illustrated by its exploitation for routine clinical diagnostics, cell therapeutic interventions and scientific study in the context of immunology, hematology and oncology [2]. The entire hematopoietic lineage has been rather well defined [3]: combinatorial codes of surface antigens are applied to define the stem, progenitor and differentiated subsets derived from hematopoietic stem cells. More than a dozen cluster of differentiation (CD) antigens are used to identify immunologically relevant subsets such as cytotoxic T-cells (positive for CD45, CD3, CD8), for instance, or hematopoietic stem cells (lineage-negative for CD2, CD3, CD11b, CD14, CD15, CD16, CD19, CD56, CD123, CD235a markers; negative for CD38, CD90; positive for CD34, CD49f). Apart from the hematopoietic pedigree, other cell lineages and tissue types have not been characterized with regard to their surface molecular signatures to the same extent. However, the recent advent of pluripotent and multipotent stem cell biology and the associated hope of timely clinical translation demands the detailed characterization of differentiation stages and lineages for a range of tissue types, and the isolation of well-defined cell subsets for *in vivo* (transplantation) as well as *in vitro* (e.g. disease modeling, screens) applications [4,5]. In a number of fields, stem cell differentiation protocols are inefficient. Enrichment is necessary as unwanted cell types need to be excluded before the *in vitro*-generated cell cultures can be used in regenerative cell replacement approaches to circumvent tumorigenic risk. Also, for *in vitro* applications the generation of purified populations is essential to exclude confounding effects of unwanted cellular contaminants that may mask effects on the target population in pharmacological or toxicity assays [6,7]. Approaches to eliminate unwanted cells include the use of cytotoxic antibodies [8], anti-mitotic reagents [9] or the introduction of suicide genes [10]. Instead, it may be advantageous to exploit surface markers to eliminate unwanted cells and to enrich the target population from a mixed, viable, genetically unmodified cell suspension. Unfortunately, due to limited characterization, surface molecule codes are frequently not available to isolate the cell types of interest by fluorescence-activated cell sorting (FACS), or by immunomagnetic cell separation. Moreover, the quantitative opportunities that flow-based cytometry can offer have not been routinely applied in neurobiology and other emerging stem cell fields. To fully realize the potential of stem cell research for basic studies and biomedical applications, additional markers are needed. Working in the context of basic as well as translational neural stem cell biology, we set out to devise a methodological strategy to identify as yet unknown surface molecular signatures of neural lineage differentiation.

**Figure 1:**
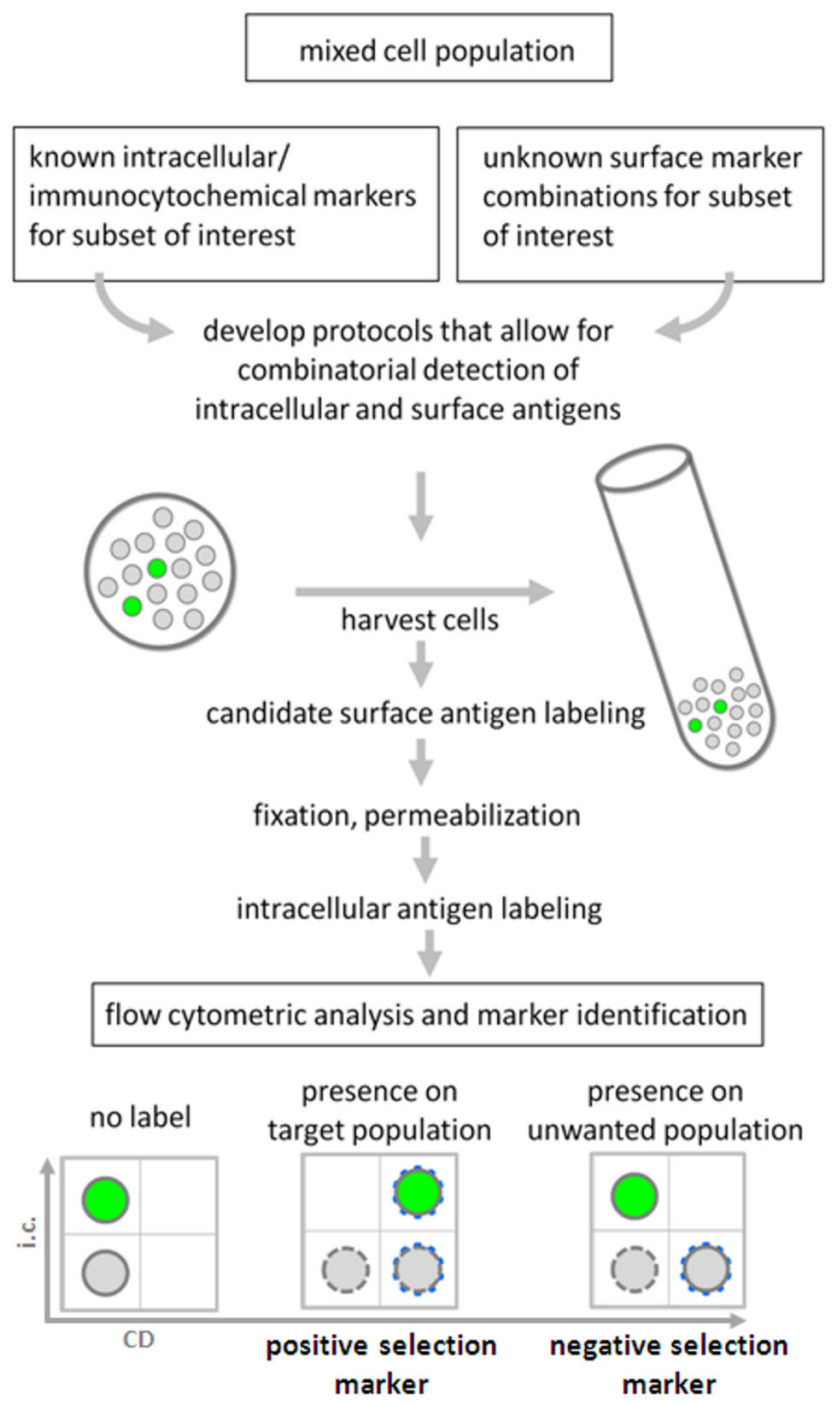
Experimental outline. Schematic illustrating the research strategy of identifying novel surface marker combinations on a target population in neural and other stem cell differentiation systems for which intracellular, standard immunocytochemical markers are well established. Following harvesting, the resulting single cell suspension is subject to surface antigen candidate staining, followed by gentle fixation, permeabilization and subsequent co-staining with known intracellular markers. CD markers co-labeling the target population serve as positive markers, those absent on the target population serve as negative markers. In a separate, subsequent step, a combination of the identified positive and/or negative CD markers enables the flow cytometric enrichment of the viable population of interest from a heterogeneous cell suspension for further study and biomedical applications.

A previously successfully applied targeted approach to identify novel surface markers and surface marker combinations is the application of genetic markers such as green fluorescent protein (GFP) under a known key promoter in combination with co-staining for a panel of CD marker candidates. We previously exploited nestin-GFP and *Sox1*-GFP reporters to identify novel neural stem cell markers and synapsin-GFP or *Pitx3*-GFP to identify mature neuronal and dopaminergic markers in pluripotent stem cell paradigms [11–13]. Co-expression of the surface marker candidate on the green fluorescent target population was used to define markers for positive selection. Conversely, surface marker candidates present on GFP-negative, unwanted cellular subsets were deemed suitable for negative selection. Similar strategies have been applied in other areas of stem cell biology and regenerative medicine. In the context of muscular dystrophy, for instance, genetic labeling for *Pax3*-GFP has been explored to define and isolate muscle satellite cells, which subsequently yielded a panel of surface marker candidates in mice [14]. In attempts to supplement lost heart tissue with cardiomyogenic cells, the quest for surface markers has recently yielded a few promising candidate markers by exploiting surface marker colocalization on *NKX2-5*-GFP-positive [15] or cardiac troponin-T (TNNT2)-immunoreactive cells in human pluripotent stem cell cultures [16]. As the generation of reporter lines is a labor-intensive and cumbersome endeavor, this somewhat defeats the purpose of utilizing the rapid analytical opportunities flow cytometry has to offer on genetically naïve, unmodified cells. Furthermore, silencing of the chosen reporter genes upon *in vitro* differentiation is not uncommon, and the selection of the most appropriate promoter may be challenging [17]. Far greater applicability of the above principle of co-labeling unknown marker candidates with established markers could be achieved if the extensive range of established stage- and lineage-specific intracellular markers routinely used in the immunocytochemical analysis of the population of interest could be exploited in an analogous manner. For many research areas, there is a stark contrast between the multitude of well-established intracellular immunocytochemical antigens and the lack of appropriate surface markers or recognized surface marker combinations that would enable the ready isolation of the population of interest. As is certainly the case for a number of (stem) cell biological research areas, a range of intracellular markers is very well established from decades of immunohistochemical and immunofluorescence work on tissue sections and primary cultures. Consequently, a feasible sequential approach could be to (1) co-stain surface antigens on intracellularly labeled cells; (2) subsequently deduce and apply the identified surface markers alone on unfixed cells; (3) validate the resulting surface marker code in neural cell sorting experiments (Figure 1). We decided to pursue this route.

In contrast to genetic fluorescent markers, flow cytometric, antibody-based analysis of intracellular antigens requires fixation and permeabilization, during which cells die. The option of sorting live cell populations for further *in vitro* culture assays or for transplantation is therefore lost. The few published protocols for intracellular staining of neural cells mostly apply either Triton X-100 or saponin-based permeabilization after standard 2 to 4% paraformaldehyde (PFA) fixation [18,19]. In addition to the resulting unavoidable cell death, CD surface antigen staining is usually severely affected or entirely lost [20,21], preventing the opportunity for co-expression analysis of CD antigens with known intracellular antigens. We set out to develop a working protocol that would enable the simultaneous detection of CD surface molecules with known intracellular markers, with the purpose of yielding neuronal differentiation markers in mammalian cell culture systems.

## Materials and Methods

### Cell lines, media and culture conditions

For most experiments, SH-SY5Y cells (ATCC CRL-2266™), originally subcloned from SK–N–SH neuroblastoma culture, were used. Cells were cultured in DMEM/F12 medium (Life Technologies) supplemented with 15% fetal bovine serum (FBS; Life Technologies), 1% non-essential amino acids (NEAA; Life Technologies) and Penicillin-Streptomycin (Life Technologies). In addition, the BE(2) M-17 cell line (ATCC CRL-2267™) was cultured in DMEM/F12 medium supplemented with 10% FBS and Penicillin-Streptomycin. As a glial control the SNB-19 line (DSMZ, ACC-325) of glioblastoma origin was cultured in DMEM medium supplemented with 10% FBS and Penicillin-Streptomycin. As a negative, non-neural cell control BJ fibroblasts (ATCC CRL-2522™) were used. This line was cultured in α-MEM medium (Life Technologies) supplemented with 10% FBS and Penicillin-Streptomycin. For iPS cell generation, dermal skin fibroblasts were obtained from healthy controls and informed written consent was obtained from the subjects involved in our study prior to cell donation. The Ethics Committee of the Medical Faculty and the University Hospital, Tübingen approved this consent procedure and this study. Fibroblast medium consisted of DMEM (Life Technologies) supplemented with 10% FBS, 1% penicillin/streptomycin/glutamine, 1% NEAA, 1% sodium pyruvate (all PAA), and 0.5 mM β-mercaptoethanol (Life Technologies). Reprogramming and characterization of iPS cells [22] and neural differentiation [23] were performed as previously described. Cells were used for analysis at day in vitro (DIV) 45 to 75. All cultures were maintained at 37 °C in a 5% CO_2_ humidified incubator with media being changed every one to three days.

### Harvesting

Cells were harvested and gently dissociated using TrypLE (Life Technologies) to obtain a single cell suspension of 0.5–2 × 10^6^ cells per mL as previously described [13]. Cell viability ranged above 90% as determined by trypan blue dye exclusion. Cells for flow cytometric analysis were taken up in Ca^2+^/Mg^2+^-free phosphate buffered saline (PBS; PAA Laboratories) with 2% FBS and distributed into 1.5 mL microcentrifuge tubes for subsequent experimental steps. Centrifugation steps were conducted using a refrigerated table microcentrifuge (Peqlab Perfect Spin 24 R) at 2400 rpm (542 g) for three to five minutes.

### Fixation and Permeabilization

**Figure 2:**
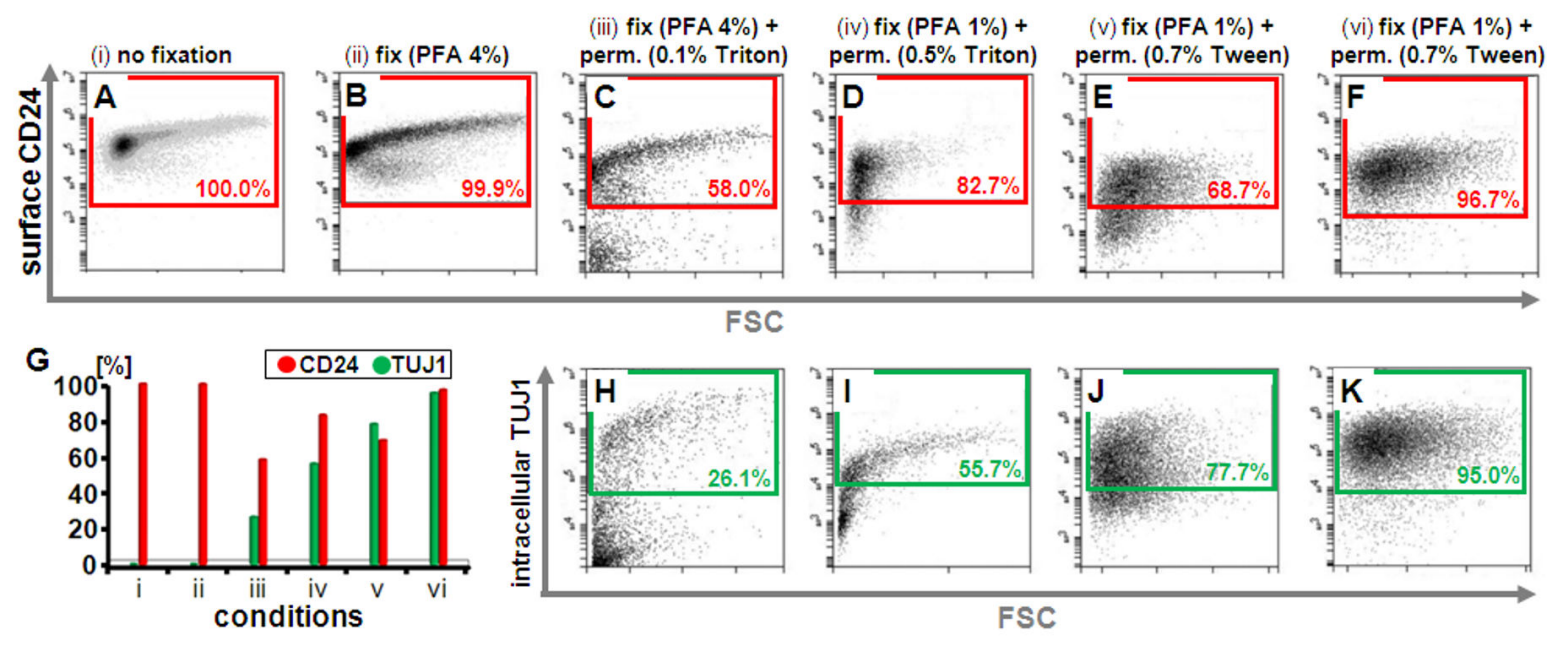
Establishing conditions that maintain CD surface marker labeling with detection of intracellular antigens. CD24 surface antigen detection on SH-SY5Y cells remained largely stable with 4% PFA fixation after staining (**A**, **B**), yet was greatly reduced with permeabilization using Triton X-100(C, D). Lowering PFA concentration combined with Tween-20 for permeabilization restored CD24 surface staining to levels approximating those seen on live cells (**E**, **F**; see **A**). Optimizing fixation and permeabilization (**H** to **K**) enabled simultaneous detection of intracellular antigens, here TUJ1 (β-III-tubulin) while preserving cell surface staining (see **F**, **K**). Panel (**G**) provides a quantitative overview of the different conditions (i-vi indicating the conditions as labeled in **A** to **F**). Incubation of live or solely fixed cells with antibodies targeting intracellular epitopes without permeabilization results in no detection. Representative scatter plots for ≥ three independent experimental repeats are shown.

Solutions used in the following protocol were prepared in PBS at concentrations as indicated in Figures **2** and **4**. PFA (Carl Roth, GmbH) was used at concentrations of 0.5 to 4% (w/v). Triton X-100 (Sigma) and Tween-20 (Sigma) were added at concentrations ranging from 0.1 to 0.7% (v/v).

### Antibodies

Antibodies used to detect intracellular and surface antigens are listed in Tables **1** and **2**, respectively. All antibodies were carefully titrated. Direct conjugates were used to detect surface antigens. Intracellular antigens were detected by Alexa Fluor secondary antibodies (Life Technologies) as indicated. Wherever available, isotype-specific antibodies were used for secondary detection. Dilutions of conjugated surface antibodies were as listed in Table **1**, ranging from 1:25 to 1:50. While concentrations may vary with each lot and were not specified by all providers, these dilutions largely correspond to final concentrations in the range of 0.5 to 4 µg/mL.

**Table 1:** Compilation of intracellular primary and secondary antibodies used, including optimized dilutions and concentrations.

	**Antibody**	**Reactivity**	**Host**	**Dilutions**	**Manufacturer**	**Clone/Isotype**	**Stock Concentration**	**Final Concentration**
**Intracellular Antigens, primary antibodies**	TUJ1	Mammalian	Rabbit	1:2000	Covance	-----	1 mg/mL	0.5 µg/mL
	DCX	Human, mouse, rat	Goat	1:500	Santa Cruz	C-18/IgG	0.2 mg/mL	2 µg/mL
	MAP2	Human, mouse, rat	Mouse	1:500	Millipore	AP20/IgG1	1 mg/mL	0.4 µg/mL
	Nestin	Human	Mouse	1:1000	Neuromics	196908/IgG1	1 mg/mL	1 µg/mL
	GFAP	Human, cow, mouse, rat,	Rabbit	1:500	Dako	-----	2.9 mg/mL	5.8 µg/mL
	TH	Mammalian	Rabbit	1:200	Pel-Freez	-----	-----	-----
**Secondary antibodies**	Alexa Fluor 488	Rabbit	Donkey	1:2000	Life Technologies	- / IgG	2 mg/mL	1 µg/mL
		Mouse	Donkey	1:1000	Life Technologies	- / IgG	2 mg/mL	2 µg/mL
	Alexa Fluor 647	Goat	Donkey	1:2000	Life Technologies	- / IgG	2 mg/mL	1 µg/mL
		Mouse	Goat	1:1000	Life Technologies	- / IgG	2 mg/mL	2 µg/mL
		Rabbit	Goat	1:2000	Life Technologies	- / IgG	2 mg/mL	1 µg/mL

Green or far-red fluorescence were used for intracellular antigen detection (Alexa Fluor-488 or -647)

**Table 2:** Compilation of CD surface antigen antibodies used, including optimized dilutions and concentrations.

	**Antibody**	**Reactivity**	**Host**	**Dilutions**	**Manufacturer**	**Clone/Isotype**	**Stock Concentration**	**Final Concentration**
**Surface markers**	CD24 PE	Human	Mouse	1:50	eBioscience	eBioSN3 / IgG1	0.05 mg/mL	1 µg/mL
	CD29 PE	Human	Mouse	1:33	eBioscience	TS2-16/IgG1	0.025 mg/mL	0.75 µg/mL
	CD49f PE	Human, mouse	Rat	1:50	eBioscience	eBioGoH3/IgG2a,к	0.2 mg/mL	4 µg/mL
	CD56 PE	Human	Mouse	1:33	eBioscience	CMSSB/ IgG1,κ	0.025 mg/mL	0.75 µg/mL
	CD71 APC	Human	Mouse	1:50	eBioscience	OKT-9/ IgG1	0.012 mg/mL	0.24 µg/mL
	CD90 APC	Human	Mouse	1:50	eBioscience	eBio5E10 / IgG1,κ	0.05 mg/mL	1 µg/mL
	CD133 APC	Human	Mouse	1:33	Miltenyi	AC133 / IgG1	0.05 mg/mL	1 µg/mL
	CD184 APC	Human	Mouse	1:50	eBioscience	12G5 / IgG2a,κ	0.05 mg/mL	1 µg/mL
	CD200 APC	Human	Mouse	1:50	eBioscience	OX104 / IgG1	0.05 mg/mL	1 µg/mL
	CD271 Alexa Fluor 647	Human	Mouse	1:33	BD	C40-1457/IgG1,κ	-----	-----

Phycoerythrin- or Allophycocyanin-conjugated antibodies were used for surface molecule analysis

### Intracellular Antigen, Staining

The resulting optimized protocol is detailed below (see narrative). In brief, cell suspensions were stained with conjugated antibodies detecting surface antigens followed by fixation, permeabilization and subsequent indirect staining for intracellular antigens. Primary antibodies were incubated in the dark with 10% normal goat (PAA) or donkey (Millipore) serum and 1% bovine serum albumin (PAA) in PBS. For washing steps the supernatant was removed by decanting, allowing a minor volume of liquid (ca. 100 µL) to remain. Note that a considerable amount of cell loss is to be expected due to repeated washing. Adherence to the recommended initial cell concentrations (0.5 to 2 x 10^6^ cells per mL) is needed to ensure analytical quality and readout of >3,000 cells within the overall population gate to be analyzed.

### Flow Cytometry

Flow cytometric analysis conducted immediately after staining consisted of two to three color panels. We acquired data on a BD Accuri C6 benchtop cytometer equipped with FL1 (533/30), FL2 (585/40) and FL4 (675/25) bandpass filters. Data were analyzed and are presented using BD CFlow software version 1.0.227.4. Flow cytometry screening raw data is available at flowrepository.org. Neuronal cell sorting was performed on BD FACSAria III or on Beckman Coulter MoFlo instruments using a 100 µm nozzle and sheath pressure of 20 to 25 PSI as previously described in detail [13]. Excluding debris and the minor amount of cells observed to be dead after harvesting, a primary gate based on forward and side scatter was set to select the overall population of interest (designated as “live gate” for naïve viable cells; “overall population gate” for fixed/permeabilized cell suspensions). Viability of these populations was assessed by fixable LIVE/DEAD assay (Life Technologies) to routinely range above 99% of cells, and FSC-peak (height) versus FSC-integral (area) gating was applied to exclude doublets for cell sorting. Gates for detecting positive staining were set against unstained controls for surface antigens. Fluorescence gates for intracellular antigen detection were set to 0.4 or 0.9% by acquiring controls in which the primary antibody had been omitted. Where appropriate, compensation was applied according to single-stained controls of the same cell type included in each individual experiment.

### Statistics

Quantification is shown of at least three independent experimental repeats. Student’s t-test was used to determine significance. A *p*-value below 0.05 was considered statistically significant. Error bars indicate standard deviation.

## Results

### Preservation of CD surface antigens with detection of intracellular antigens

One of the critical issues encountered in the intended approach is that the permeabilization and fixation steps required for intracellular antigen detection negatively affect or completely ablate surface antigen staining. This was exemplified with the known neural differentiation marker CD24 under live, fixed, and fixed-and-permeabilized conditions on SH-SY5Y neuroblastoma cells (Figure **2A–E**; G-J; Figure **S1**). While fixation using standard conditions with 4% PFA only marginally affected prior CD surface marker staining, permeabilization with non-ionic detergents routinely used in immunocytochemical assays (Triton X-100; Tween-20) or in flow cytometry protocols for sole detection of intracellular antigens (e.g., the amphipathic glycoside saponin) was found to have a negative effect on the ability to detect a wide range of surface molecular epitopes (Figure **S2**). Ultimately, a combination of 1% to 2% PFA fixation with 0.5 to 0.7% of the detergent Tween-20 (Polysorbate 20; polyoxyethylenesorbitan monolaurate) for the permeabilization step was determined to enable the preservation of CD surface antigens while enabling the detection of intracellular antigens (Figure 2**F,G**,**K**).

### *Validation of intracellular detection specificity in this protocol*

**Figure 3:**
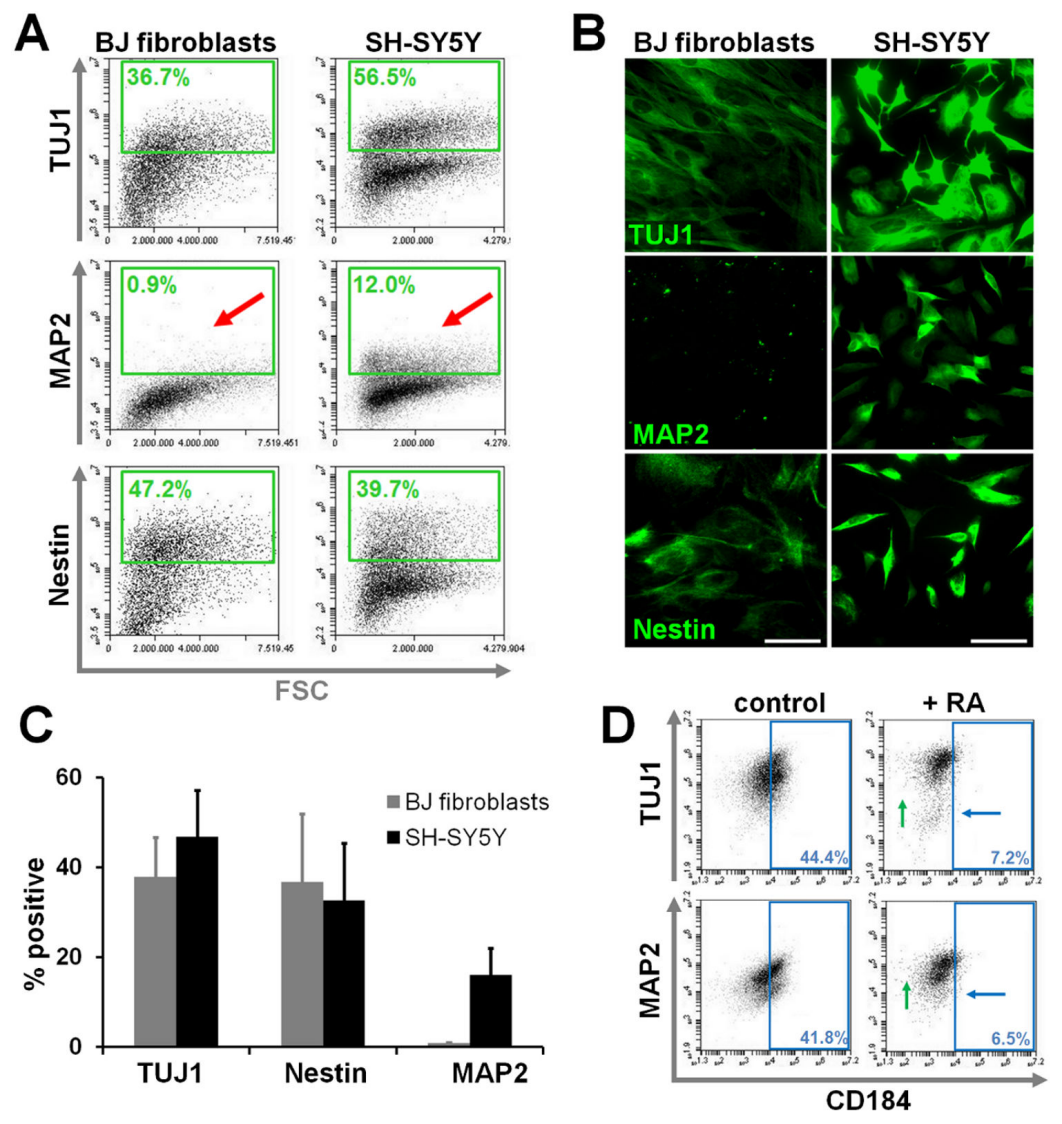
Accurate detection of intracellular antigens with optimized fixation-permeabilization conditions preserving surface antigens. Flow cytometric detection of TUJ1, MAP2 and nestin antigens in BJ fibroblasts and the neural SH-SY5Y cell line (A). TUJ1 and nestin are present in both cell lines, while the mature neuronal marker MAP2 was only detected in SH-SY5Y cells (arrows). Note stable fluorescent levels of the negative population, indicating low background staining using this protocol. Representative experiment of three independent repeats shown. (**B**) Corresponding validation by immunofluorescence analysis. (**C**) Quantitation of TUJ1, MAP2 and nestin intracellular antigen detection (n=3). Error bars indicate standard deviation. (**D**) Response of TUJ1 and MAP2 intracellular antigen expression to 6 DIV of 10 µM retinoic acid (RA) treatment of SH-SY5Y cells. Note disappearance/reduction of subsets negative for these markers (upward shift, green arrows), as well as a shift toward CD184^low^ expression with differentiation (blue arrows).

Having established conditions that allowed for preservation of surface epitope detection with fixation and permeabilization steps, we next intended to document accuracy of the intracellular stain and its applicability in quantitative assays. A stringent method to demonstrate specificity of antigen labeling is the absence of detection in an unrelated cell type known to be deficient for that particular antigen (“cell controls”). To this end, we compared the SH-SY5Y cell line, that is able to undergo neuronal differentiation, to the human foreskin-derived BJ fibroblast line. Detection of neuronal-specific microtubule-associated protein-2 (MAP2) was limited to SH-SY5Y cells able to undergo neuronal differentiation, while other neural micro- and intermediate filament markers (β-III-tubulin [TUJ1 antigen]; nestin) known to be present in fibroblasts [24–27] were also detected in the BJ line (Figure 3A,B). Titrations of primary and secondary antibodies were performed to achieve distinct separation of positive versus negative fractions and gates were set against controls in which the primary antibody had been omitted. We noted that dilutions of 1:1000 to 1:2000 of the fluorescent secondary antibody could greatly diminish background fluorescence. As a further indicator of optimized staining specificity, fluorescence levels of the negative fraction present in either cell type remained at levels comparable to the corresponding controls (i.e. 2° antibody only; between 10^2^ to 10^4^ on arbitrary logarithmic scale). MAP2 numbers in SH-SY5Y cells ranged from 10 to 20%, while flow cytometric immunoreactivity for TUJ1 and nestin was around 40% (Figure 3C) in accordance with immunocytochemical detection (see Figure 3B) and published results [28,29] and responded appropriately to differentiation stimuli (retinoic acid treatment of SH-SY5Y cells; Figure 3D).

**Figure 4:**
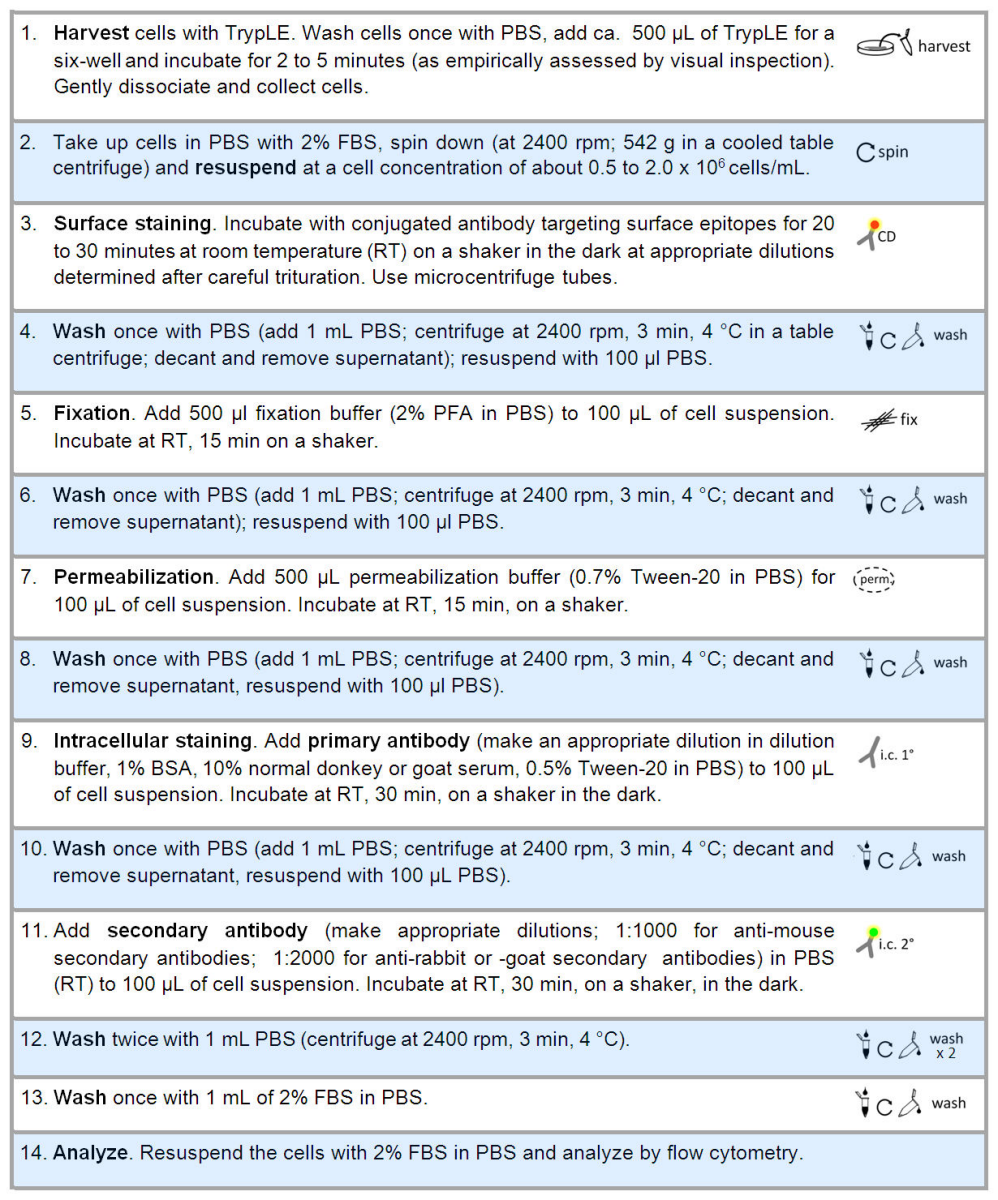
Optimized protocol. The detailed list of steps illustrates the working protocol for combined surface and intracellular epitope staining of mammalian cells for flow cytometric analysis. See methods section for further details on reagents and procedures.

### *Application in comparative intracellular antigen analysis*

**Figure 5:**
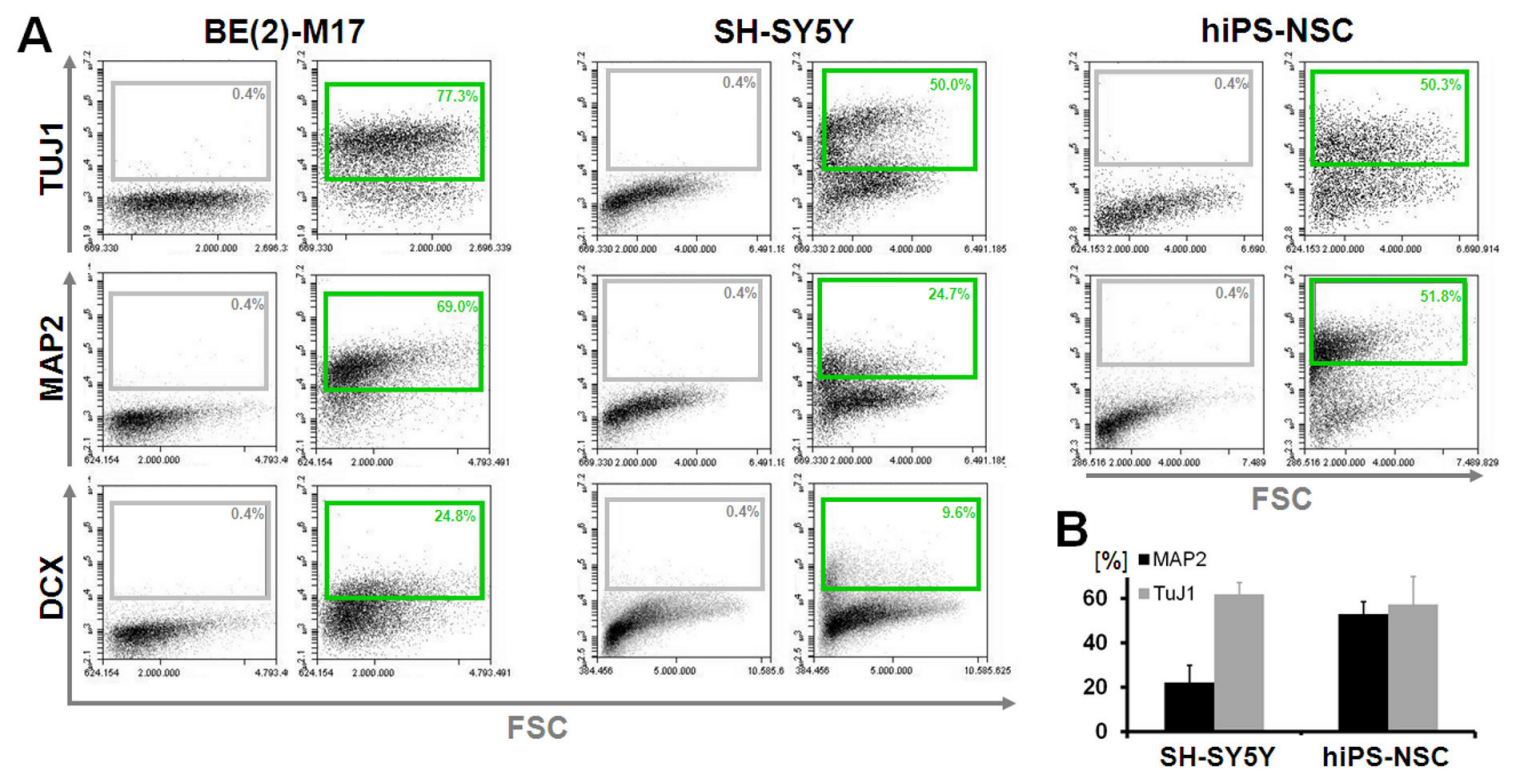
Qualitative and quantitative comparison of intracellular markers expressed by neural cells. (**A**) Flow cytometry for the intracellular antigens TUJ1, MAP2 and doublecortin (DCX) analyzed on various cell sources including neuroblastoma BE(2)-M17 and SH-SY5Y as well as human iPS cell-derived differentiated neural stem cell cultures (hiPS-NSC). Grey boxes in flow cytometry plots specify gates set on negative control samples to capture 0.4%. Green boxes specify positive fraction on stained samples. Error bars in (**B**) indicate standard deviation.

Next, we applied the resulting protocol (Figure 4) on a range of human cell lines capable of neuronal differentiation including the SH-SY5Y line, the BE(2)-M17 line (originally derived from the SK–N-BE(2) neuroblastoma cell line) as well as differentiated human neural stem cell cultures generated from iPS cells (Figure 5A). Confined margins of error were observed when quantitatively analyzing data on intracellular antigen expression from three independent experimental series (Figure 5B). While TUJ1 levels were found to be present at comparable levels in SH-SY5Y and in neurally differentiated human pluripotent stem cell cultures (53.1±5.5 vs. 57.4±12.7; *p* = 0.6190), the more mature neuronal somatodendritic marker MAP2 was higher in iPS cell neural differentiation as opposed to the proliferative neural cancer culture (62.0±5.3 vs. 22.0±7.8; *p* = 0.0018). We concluded that the resulting protocol, while maintaining surface antigen detection, allowed for faithful and reproducible detection of intracellular antigens.

### *Application in CD surface marker identification*

**Figure 6:**
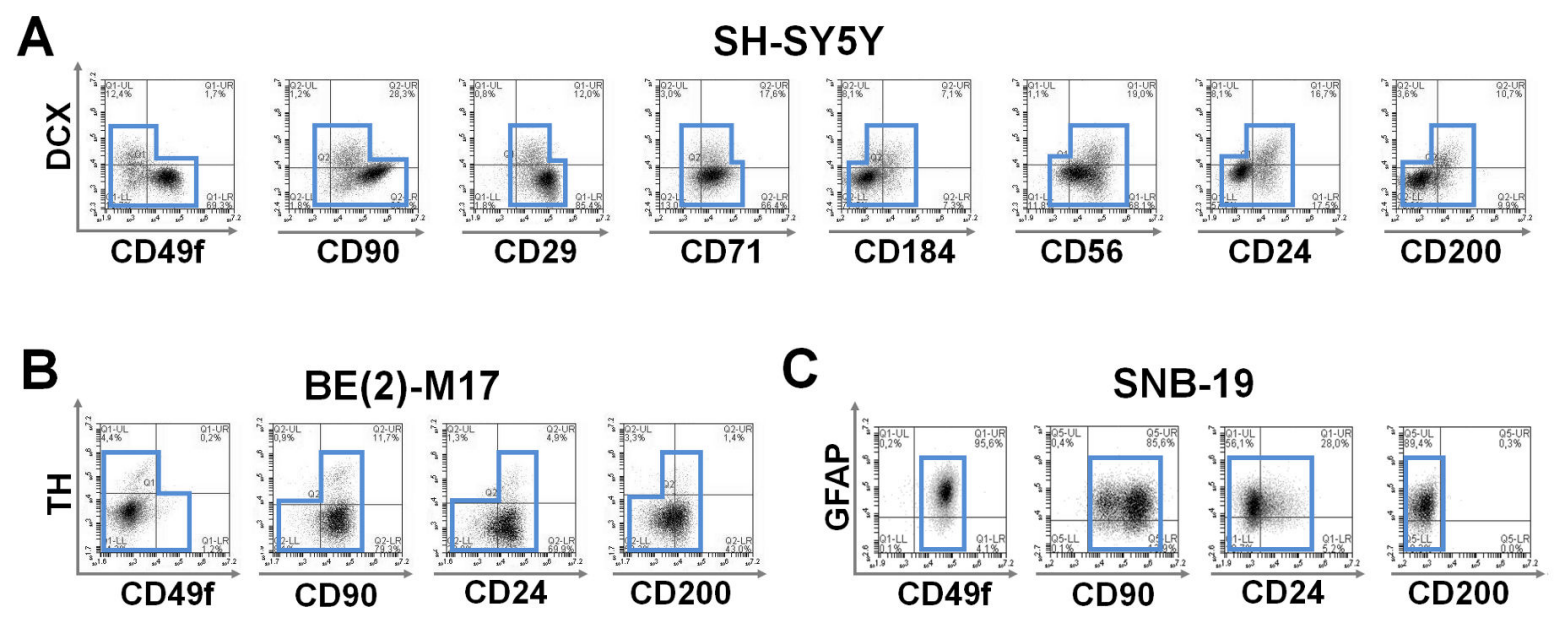
Combinatorial detection of known neural intracellular markers plus a panel of candidate surface antigens in human cell lines. (**A**) In SH-SY5Y cells CD49f, CD90^HIGH^ and CD29 expression clustered distinct from doublecortin (DCX)-positivity, while subsets of CD56, CD24 and CD200 closely correlated with neuronal differentiation (DCX+). Patterns of exclusive positivity on either the y- or the x-axis (upper left and lower right quadrant) yield CD marker candidates for negative selection strategies. Patterns of shared intracellular and surface expression (upper right quadrant) are suitable for positive selection strategies. Blue outlines illustrate co-expression patterns. (**B**) Co-localization analysis of the catecholaminergic intracellular marker tyrosine hydroxylase (TH) is shown on BE(2)-M17 cells. The TH-positive subset stained negative for the putative proliferative indicator CD49f but was colocalized with CD90, CD24 and CD200 in this cell line. (**C**) Co-localization analysis of the glial intracellular marker glial fibrillary acidic protein (GFAP) is shown on SNB-19 cells. In contrast to the other neural cell lines analyzed, GFAP-positive subsets stained positive for CD49f, and also CD90. CD24 was present to a lower degree and the putative neuronal marker CD200 in this context was virtually absent.

To demonstrate this protocol’s technical suitability for application in future screens for CD markers of cell populations of biomedical interest, we co-stained a selection of well-established intracellular neuronal differentiation antigens combined with a range of CD marker candidates on several human cell lines. Exclusive positivity on either the *y*- or the *x*-axis (upper left and lower right quadrant) without co-labeling of surface with intracellular antigen (upper right quadrant) was deemed to characterize CD marker candidates suitable for eliminating proliferative components in neuronal enrichment paradigms (*negative selection* strategy). Shared intracellular and surface expression (upper right quadrant) was deemed suitable for *positive selection* strategies. Analyzing the patterns of CD surface antigen co-expression (or absence) in SH-SY5Y cells, we identified CD49f (α6-integrin) to be negatively correlated with intracellular neuronal antigen expression and thereby largely associated with neural proliferation (Figure 6A), a pattern previously observed with its known heterodimeric partner CD29 (β1-integrin) [12,30]. We found the previously established neural markers CD56 (NCAM1, MSK39) [13] and CD24 (heat stable antigen; small cell lung carcinoma cluster 4 antigen) [12] to be correlated with neuronal differentiation, indicated by TUJ1 and/or doublecortin co-staining, in SH-SY5Y as well as in human pluripotent stem cell-derived neural cells. In addition, other CD markers found to be present on neuronally differentiating cells in human neural cancer cell cultures were the transferrin receptor protein-1 (CD71), the Thy-1 antigen (CD90), the tetraspanin family member CD151 and the membrane glycoprotein MRC OX-2 (MOX2; CD200). The latter was particularly well correlated with neuronal differentiation markers. Next, we set out to validate that these markers would enable the purification of mature neurons of biomedical interest. To this end, we used surface co-labeling of the catecholaminergic, tyrosine hydroxylase-positive subset in BE(2)-M17 cells. CD49f was confirmed as one of the previously identified *negative selection* markers for neuronal cell sorting also in this cell line. In contrast, CD90 appeared to be co-expressed in BE(2)-M17 cells on tyrosine hydroxylase-as well as on doublecortin-positive cells (Figure 6B). Moreover, using the SNB-19 line of glioblastoma origin, we found CD49f present on glial fibrillary acidic protein (GFAP)-positive cells, while CD200 was not co-expressed with this glial marker, in contrast to its close correlation with the other neural markers analyzed (Figure 6C).

Mere percentage of marker co-expression alone does not account for the possible presence of intracellular-only or CD-only single-labeled populations in addition to the co-labeled subset, which represents a potential confounding factor for CD marker-based cell isolation. Similarly, due to the heterogeneity of the overall sample (resulting in the presence of distinct subsets with varying fluorescence intensity), we determined the Pearson coefficient correlation [31] to be insufficient for the identification of new CD markers co-localized on only some of the constituent subpopulations. The most efficient isolation of the target population will occur when the identified CD marker is exclusively or preferentially expressed on the population of interest, but absent to low in terms of expression on any other subsets. To this end and to avoid arbitrary gating when defining such potential subsets, we decided to use easily standardizable quadrant gates. We devised the strategy of applying ratios of positive marker fluorescence on the intracellularly-labeled/ CD marker-negative versus that of the co-labeled subset. As an additional criterion for candidate marker selection, we defined a standardized co-expression score (CS) of (*F*_*UL*_ */F*_*LL*_) */* (*F*_*UR*_ */F*_*LR*_) *= CS*, wherein *F*_*UL*_ referred to fluorescence percentage in the upper left quadrant (+/–), *F*_*LL*_ to lower left (-/-), *F*_*UR*_ to upper right (+/+) and *F*_*LL*_ to lower right (-/+) quadrants when analyzed as shown (abscissa: CD marker; ordinate: intracellular label). Co-expression scores are dependent on the degree of positivity (percentage and mean fluorescence intensity) of the particular intracellular antigen. Thus, each individual intracellular marker required a separate percentile ranking to generate a heatmap based on co-expression scores (see Figure **S3**). Quantitatively analyzing co-expression in SY-SY5Y cells in this objective manner, CD49f and CD29 showed overall the clearest aptitude as negative markers (co-expression ratios ranging from *CS* = 0.03 to 0.8) while CD24 and CD200 qualified as positive markers for doublecortin-positive cells (*CS* = 6.8 and 22.8, respectively). From these co-expression analyses on fixed, permeabilized (non-viable) neural cell preparations of these human cell lines, we concluded that CD49f was a candidate CD marker associated with the proliferative state, while CD200 promised to bear utility for enrichment of mature neurons.

### *Exploiting the resulting markers for cell sorting of viable neural subpopulations*

**Figure 7:**
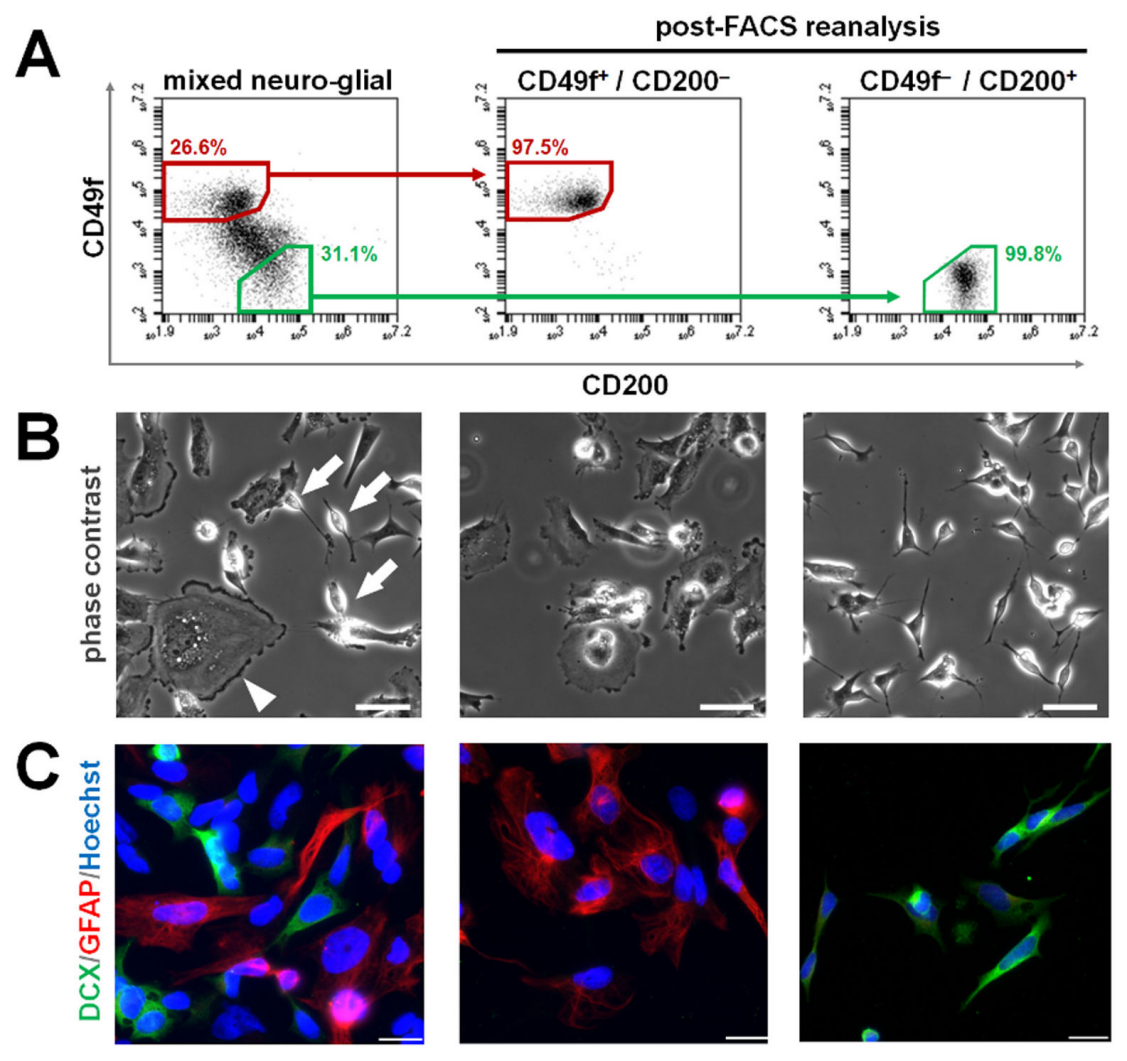
Application for neural cell isolation in human neural cancer lines. (**A**) Mixed SH-SY5Y/SNB-19 neuro-glial cell suspensions (without fixation and permeabilization) analyzed by flow cytometry for CD49f-PE and CD200-APC. Polygon gates within the left dot plot indicate selection of CD49f^+^/CD200^-^ (red) versus CD49f ^-^/CD200^HIGH^ (green) populations. (**B**) Corresponding microscopic analysis of unsorted (left panel) vs. sorted conditions (mid and right panels) 1 div post-sort. Arrows indicate SH-SY5Y cells, arrowhead SNB-19 [phase contrast; scale bars: 50µm]. (**C**) Immunofluorescence for GFAP (red) and doublecortin (green) of surface marker code-based cell suspensions. Left panel: unsorted; mid panel: CD49f^+^/CD200^-^ population post-sort; right panel: CD49f ^-^/CD200^HIGH^ population. [1 div post-FACS; scale bars: 20µm].

**Figure 8:**
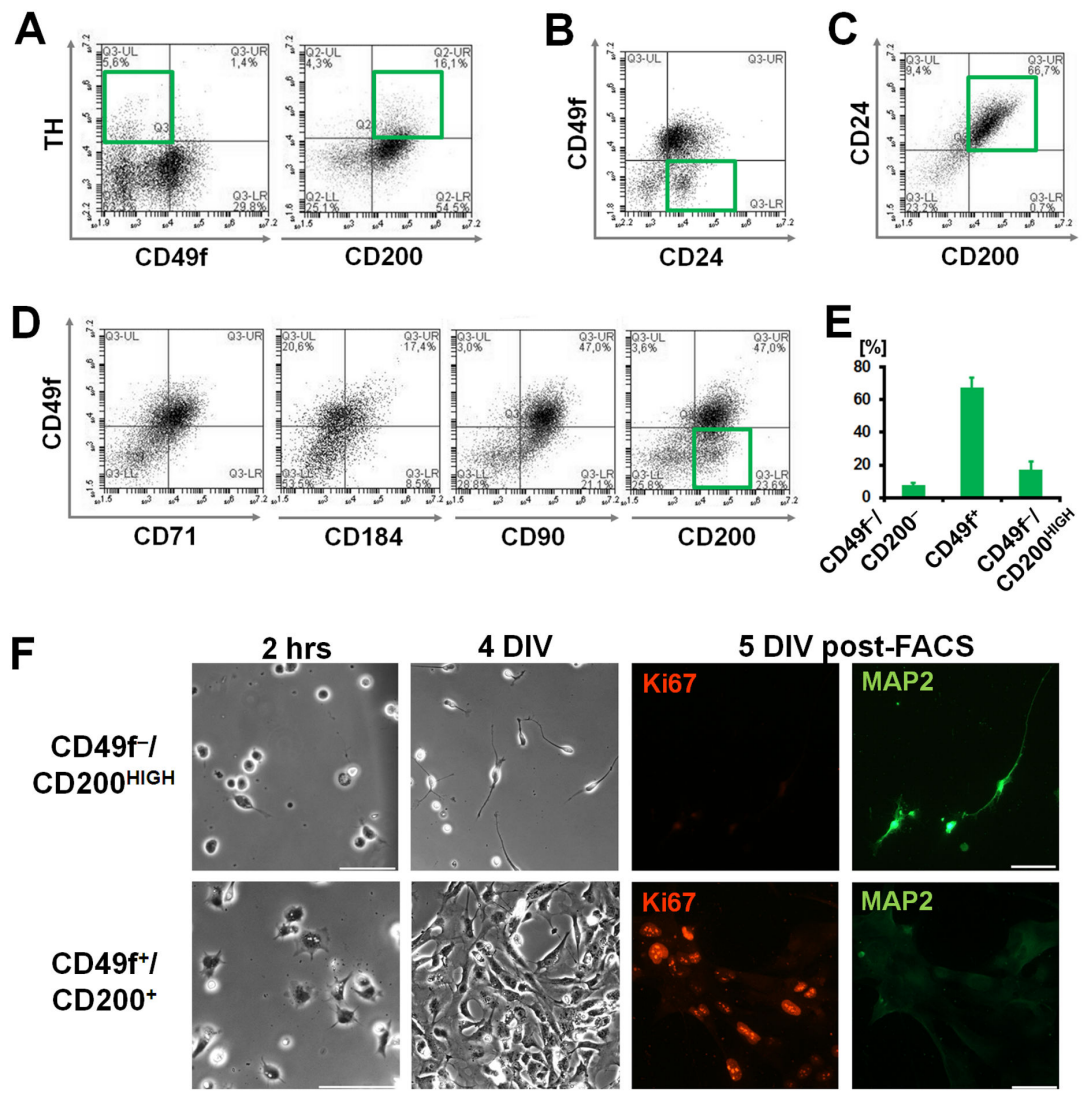
Application for neural cell isolation in human iPS cells. (**A**) Flow cytometric analysis of the intracellular neuronal subpopulation marker tyrosine hydroxylase (TH) with surface marker candidates CD49f and CD200 (on fixed and permeabilized hiPS-NSC). (**B**, **C**) Co-expression analysis of CD49f and CD200 with the established neural marker CD24. (**D**) Co-expression analysis of CD49f with CD71, CD184, CD90, CD200 antigens. Note occurrence of CD49f-negative subset with CD200 co-labeling (green rectangular gate). (**E**) Presence of CD49f/CD200 subpopulations in hiPS-NSC differentiation (four independent experimental lines). (**F**) Phase contrast and immunofluorescence imaging post-FACS; hiPS-NSC at 2 hours, DIV 4 and 5 after CD49f/CD200 FACS. [Scale bars: 50µm. Panels **B** to **D** on live hiPS-NSC suspensions without fixation and permeabilization].

Consequently, we aimed to verify the utility of CD markers resulting from our screening strategy for scientific and biomedical stem cell applications. First, as a proof-of-principle, we used live neuro-glial cell suspensions obtained by mixing equal amounts of SNB-19 and SH-SY5Y cells. Sorting for CD49^+^/CD200^-^ enabled the isolation of glial cultures from the heterogeneous parent population. In contrast, the CD49f^-^/CD200^HIGH^ post-FACS fraction was enriched for doublecortin-positive SH-SY5Y neuroblasts and was virtually devoid of GFAP-positive glial cells (Figure 7A–C), underlining the utility of the identified markers for isolating neurogenic subsets from heterogeneous neural cell preparations. Next, CD49f and CD200 were analyzed on iPS cell-derived neurally differentiated cultures. In our protocol, the catecholaminergic marker tyrosine hydroxylase can serve as an indicator of neuronal maturity and postmitotic differentiation [11,32]. CD49f was confirmed to be absent on the tyrosine hydroxylase-positive subset, while the putative *positive selection* candidate CD200 was co-expressed (Figure 8A). On live, pluripotent stem cell-derived neural cell suspensions, CD49f was absent on a subset of the CD24-positive population, a surface marker known to partially correlate with neural differentiation in this setting (Figure 8B). In contrast, CD200 was largely co-localized with CD24, underlining the potential utility of CD49f-CD200 combinatorial surface staining as neural stem cell versus neuronal differentiation markers (Figure 8C). Moreover, this combinatorial analysis also illustrated that single marker strategies would likely be insufficient, due to the presence of other, non-neuronal subpopulations in the CD49f-negative as well as in the CD200-positive subsets. Accordingly, CD49f-positive cells comprised a diverse array of populations co-expressing neural stem and other less-defined neural markers (e.g., CD71, CD184, CD90; Figure 8C). Combining CD49f with CD200 detection, the occurrence of a CD200-positive, CD49f-negative population became apparent in the lower right quadrant (Figure **8D**, far right panel). The characteristic pattern of a CD49f-positive (67.5±6.2%), a double-negative (8.0±1.0%) and the CD200^HIGH^/CD49f-negative (16.9±5.3%) neuronal subset was consistently encountered in human pluripotent stem cell-derived neuronal differentiation culture (four independently derived iPS cell lines; DIV 45 to 75) (Figure 8E). Finally, combinatorial CD49f-CD200 surface staining was applied in FACS, clearly enriching for neuronally differentiated (MAP2) versus proliferative (ki67) subsets in neuronally differentiated human pluripotent stem cell-derived cultures (Figure 8F).

## Discussion

We present a protocol for combined intracellular and surface antigen staining to identify novel cell surface molecular signatures on subpopulations of neural cell types (see Figure 4). Moreover, we provide the template for the identification of urgently needed CD surface marker combinations on target populations of interest which can serve as a model approach for a wide range of (stem) cell differentiation and tissue systems. Methodologically, we present a simple protocol optimized for flow cytometric quantitation of intracellular neural antigens including nestin, GFAP, doublecortin, TUJ1, MAP2 and tyrosine hydroxylase. Intracellular staining protocols and commercially available kits have been optimized for hematological/immunological paradigms to investigate signaling pathway components (e.g., phosphoproteins), cytokines and other phenotype-characteristic enzymes (e.g., myeloperoxidase [21]). However, no particular intracellular staining protocol is equally well-suited for all cell types or for all intracellular antigens. As damage to some epitopes by fixation can occur [20] we decided to perform surface staining prior to fixation-permeabilization (see Figure **S1**). This critical step should not enhance autofluorescence, avoid cell damage and reduce as much as possible the perceived high variability associated with intracellular staining due to unspecific incorporation of antibody complexes and binding to surface and intracellular epitopes [21,33]. Our results suggest that a combination of low concentration of crosslinking, non-coagulant fixative (e.g., PFA concentrations ranging from 1 to 2% for 5 to 30 minutes at 4 to 21°C) with a non-ionic detergent as described here in detail, may generally yield protocols that enable the preservation of a range of surface epitopes for combinatorial analysis and marker identification without the use of commercial kits. While nuclear antigens may be more amenable to saponin-permeabilization, this negatively affects surface antigens to a higher degree, and enhanced background fluorescence has been observed with saponin as opposed to Tween-20 (data not shown and [34]). Moreover, after saponin-permeabilization, profound changes of forward and side scatter patterns have been observed with a particular loss of forward scatter signal intensity [35].

To determine specific fluorescence signal, it is critical to make use of the most appropriate controls. While certain analyses, e.g. on B-lymphocytic populations and other Fc receptor-bearing cells, may warrant the application of isotype controls, this is not necessary or advantageous for most settings [36]. Secondary antibody-only controls are essential in indirect staining protocols, and wherever possible, the inclusion of “cell controls” is recommended (see Fig. 3). Using such controls, the definition of objective cut-off values for positivity of, e.g. <0.5 or <1.0% fluorescent events to be allowed in the gates of the appropriate negative controls, is critical. Determining the cut-off values between positive and negative populations may become a particular problem with weakly-reacting antibodies due to poor separation and, consequently, the fluorescence of the positive overlapping with the negative populations. As background fluorescence in flow cytometric intracellular staining protocols is largely dependent on primary and secondary antibodies, nonspecific binding or interference with cell surface antibodies needs to be minimized in the combinatorial intracellular/surface labeling approach. Use of conjugated antibodies decreases the possibility of nonspecific binding or of trapping of antibody complexes within the cell. Moreover, while for immunological/hematological paradigms conjugated intracellular antibodies are more readily available, this is not the case for a range of antigens in neural differentiation. Thus, we present an approach focusing on indirect (primary plus secondary antibody) detection of intracellular neural epitopes. When selecting potential primary antibodies, established use of antibodies targeting cytoplasmic epitopes in immunocytochemistry can be a good indicator, as those used for Western blot may only recognize denatured epitopes. Polyclonal antibodies may have a higher background level, and use of monoclonal antibodies is preferable. Use of affinity-purified, cross-adsorbed (i.e., against immunoglobulin of species other than the target species) secondary antibodies may further enhance staining specificity. Unspecific binding and cross-reactivity can be blocked by using plasma or serum for blocking. We routinely applied 10% normal donkey or goat serum jointly with 1% BSA, and observed satisfactory blocking effects.

With regard to neurobiology, flow cytometric detection of intracellular antigens in neural cell systems has been introduced by Sergent-Tanguy et al. using rat primary cells [18] and, using the marker panel of GFAP, nestin and β-III-tubulin, was subsequently applied to the characterization of mouse embryonic stem cells undergoing neural differentiation [19,37]. These approaches used 0.5% saponin permeabilization and antibody incubation with 0.1% saponin in PBS. Consequently, no parallel surface stain was possible. In addition, reported background fluorescence was in the range of 5%. Here, we significantly expand the validated neural intracellular panels (adding MAP2, doublecortin, tyrosine hydroxylase) and introduce the approach of screening for surface markers that are co-expressed on a specific neural cell subset of interest defined by these intracellular markers.

Utilizing this approach, detecting intracellular antigens while preserving surface marker antigens, we identified the type-1 membrane glycoprotein CD200 (OX-2, MRC, MOX1/2), a member of the immunoglobulin superfamily, as a marker associated with neuronal differentiation of human pluripotent stem cells. In contrast, the integrin α6-subunit CD49f was found to be present on the proliferative component in these cultures. Interestingly, CD200 was also found to be absent on the SNB-19 glioblastoma line, while CD49f was highly expressed. Combining these two surface markers with one another yielded highly enriched neuronal cultures after FACS of the CD49f^-^/CD200^HIGH^ subset. Many CD markers are by no means exclusive to certain stages or lineages, and it should be noted that CD200 is promiscuously expressed in a number of tissues including neural tissue [38], mesenchymal cells [39] and tumor lines and also immature pluripotent stem cells (not shown). In addition, our own data underlines the necessity of using combinatorial marker codes as CD49f is seen to be expressed on multiple subpopulations of iPS cell-derived neural cultures (see Figure 8D). Also, CD49f has recently been described to be present on pluripotent stem cells at the immature stage [40]. Consequently, the marker set of CD49f and CD200 should also be combined with additional pluripotency markers such as Tra-1-60 or SSEA-3/-4, if rare pluripotent contaminants are to be avoided under all circumstances, for definition of neural stem cells and neurons in clinical cell transplantation. For *in vitro* purposes, the isolation of CD49f-/CD200^HIGH^ cells from neurally differentiated pluripotent cultures yielded cultures of neuronal character at high purity levels suitable for *in vitro* assays (see Figure 7). In this manner, the identified combination may represent a valid two-marker alternative to more complex marker codes such as CD15^-^/CD29^LOW^/CD24^HIGH^ [12], CD184^−^/CD44^−^/CD15^LOW^/CD24^+^ [7] or the combined CD15^-^/CD29^LOW^/CD24^HIGH^/CD44^−^/CD184^−^ (unpublished data) to identify and sort neurons from heterogeneous human stem cell cultures. Further characterization will be required to determine the possible subsets of neurons that may be preferentially enriched in the one or other paradigm. The β1-integrin CD29 has previously been identified as a marker of human neural stem cells [12,30]. Its heterodimeric partner CD49f, was not reported as part of a recent large-scale neural surface antigen screen by Yuan et al. [7]. Its functional relevance for neural stem cell niche adherence and self-renewal in conjunction with CD29 or CD104 (β4-integrin) will be a subject of future study. Beyond its utility as a marker, CD200 interactions with its receptor CD200R are well described in the rodent myeloid system. Moreover, CD200R appears to be expressed in micro-, astro- and also oligodendroglia [41], and it will be interesting to pursue future functional studies in the context of the CD200-CD200R axis in neural or potentially neuroimmunological paradigms [42].

Finally, the CD49f/CD200 cell sorting experiments serve to illustrate the applicability of the described protocol in screens to identify novel CD surface marker combinations on (stem cell-derived) cell populations. Beyond neural stem cell biology, the identified methods and approaches enable the analytical quantitation and isolation of specific cell populations of interest, for which previously no markers were available, from mixed cell preparations. The easy-to-use strategy and technical steps can be applied in the context of isolating and enriching cells for cell replacement avenues as well as for generating purified, defined subpopulations for *in vitro* toxicity or pharmacological discovery screens. Our initial preliminary step in providing a simple but highly validated protocol should now permit more global and complex analysis using multiplexed cytometric approaches. Such assays bear exciting opportunities for expanding the options for multiparameter flow cytometry in stem cell biology [43].

In summary, we present a thoroughly validated flow cytometric protocol that enables the co-detection of CD and other surface antigens on fixed, permeabilized neural cell populations defined by intracellular staining. The identification of a CD49f-CD200 marker combination for neuronal cell sorting enables functional study of these new markers, allows for the generation of purified neuronal cell cultures, and exemplifies the utility of the described protocol for quantitative assays in mammalian neurobiology and for future surface marker detection in such co-expression screening approaches.

## Supporting Information Legends

Figure S1 Effects of fixation on surface antigen staining. Fixation may negatively affect surface epitope detection by CD antibodies. (**A**) No fixation. CD24-PE detection on live population (gated as outlined by red elliptical circumference in far left column of dot plots) reveals positivity >90%. (**B**) Standard 4% PFA fixation (15 min.) prior to surface staining reduces CD24 positivity. Note occurrence of negative population (black arrowhead). (**C**) Performing the CD antibody incubation first, then followed by fixation (4% PFA, 15 min.) improves CD antigen detection. (**D**) Adjusting the overall gate (red elliptical outline) to remove debris (red arrow heads) enables the approximation of original, live gate numbers but does not reach them (E; showing quantification of CD24-PE positivity as displayed in A to D), underlining the utility mainly for qualitative co-expression analysis (while precise CD antigen quantitation should be performed on live cells). (**F**) Immunocytochemical confirmation of reduced CD24-PE detection on PFA-fixed SH-SY5Y cultures.
(TIF)

Figure S2 Effects of fixation and permeabilization on forward and side scatter fluorescence signal. (**A**) Incubation with fixative and permeabilization buffers as indicated alters flow cytometric forward and side scatter properties (SH-SY5Y cell line). Adjusting FSC resolution (arrows) enables proper visual representation and subsequent analysis of the overall population (far right column of panels). (**B**) Forward scatter signal is particularly affected by permeabilization with either detergent. Mean fluorescence intensity (MFI) of a single representative experiment is shown. (**C**) Viability assessment within FSC/SSC-based gate using a fixable red-fluorescent live/dead labeling dye.
(TIF)

Figure S3 Co-expression scores of surface antigens present on populations of interest as defined by intracellular antigens doublecortin (DCX), β-III-tubulin (TUJ1) and glial fibrillary acidic protein (GFAP). (**A**) Co-expression scores were calculated by determining the ratio of cells (percentage) present in upper right (UR) to lower right (LR) quadrants over the ratio of upper left (UL) to lower left (LL) quadrants, where surface antigen staining was shown on the abscissa and intracellular stain on the ordinate of the respective flow plots (as applied throughout this manuscript) [Coexpression score= (UR/LR)/(UL/LL)]. A percentage value of 0.1% was assigned where no cells were present in a quadrant (see SNB-19, CD200 stain). Surface antigens co-stained with DCX were quantified on SH-SY5Y cells. Surface antigens co-stained with TUJ1 were quantified on SH-SY5Y cells and neuronally differentiating cultures derived from human iPS cells, and surface antigens co-stained with GFAP were quantified on SNB-19 cells. (**B**) Using the conditional formatting function in Microsoft Excel, a dark to light-green color scale was applied to each one of the intracellular co-stained data sets to generate co-expression heatmap (see Figure **6D**). Note differential clustering of scores for SNB-19 glial cells versus the other cell sources capable of neuronal differentiation.
(TIF)
