# Supplementary figures and images for: Combined Flow Cytometric Analysis of Surface and Intracellular Antigens Reveals Surface Molecule Markers of Human Neuropoiesis
Source: PLoS One. 2013 Jun 24;8(6):e68519. doi: 10.1371/journal.pone.0068519 (PMC3691147; doi:10.1371/journal.pone.0068519)

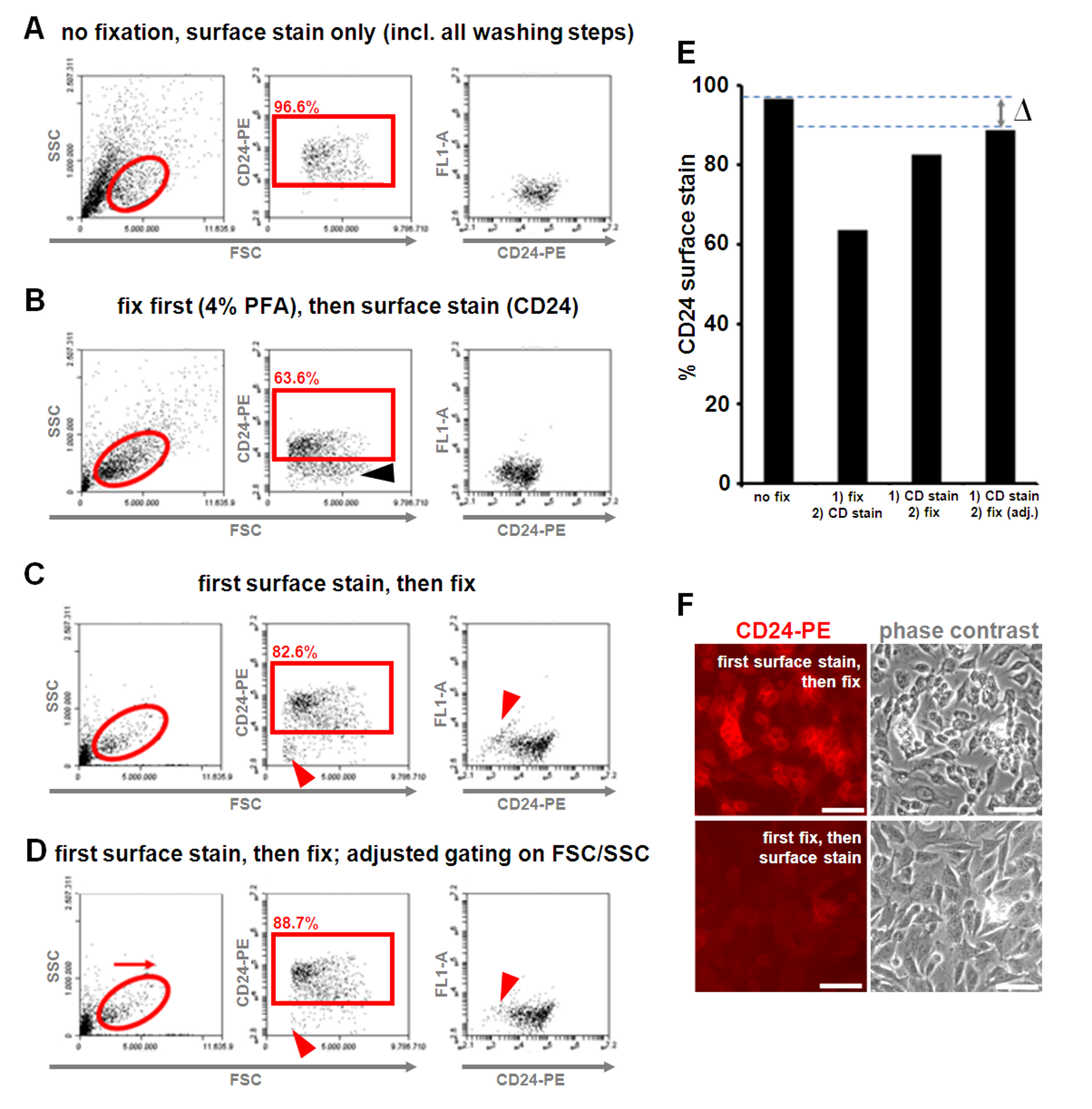

Supplement: Figure S1 — Fixation may negatively affect surface epitope detection by CD antibodies. (A) No fixation. CD24-PE detection on live population (gated as outlined by red elliptical circumference in far left column of dot plots) reveals positivity >90%. (B) Standard 4% PFA fixation (15 min.) prior to surface staining reduces CD24 positivity. Note occurrence of negative population (black arrowhead). (C) Performing the CD antibody incubation first, then followed by fixation (4% PFA, 15 min.) improves CD antigen detection. (D) Adjusting the overall gate (red elliptical outline) to remove debris (red arrow heads) enables the approximation of original, live gate numbers but does not reach them (E; showing quantification of CD24-PE positivity as displayed in A to D), underlining the utility mainly for qualitative co-expression analysis (while precise CD antigen quantitation should be performed on live cells). (F) Immunocytochemical confirmation of reduced CD24-PE detection on PFA-fixed SH-SY5Y cultures. (TIF) [file pone.0068519.s001.tif]

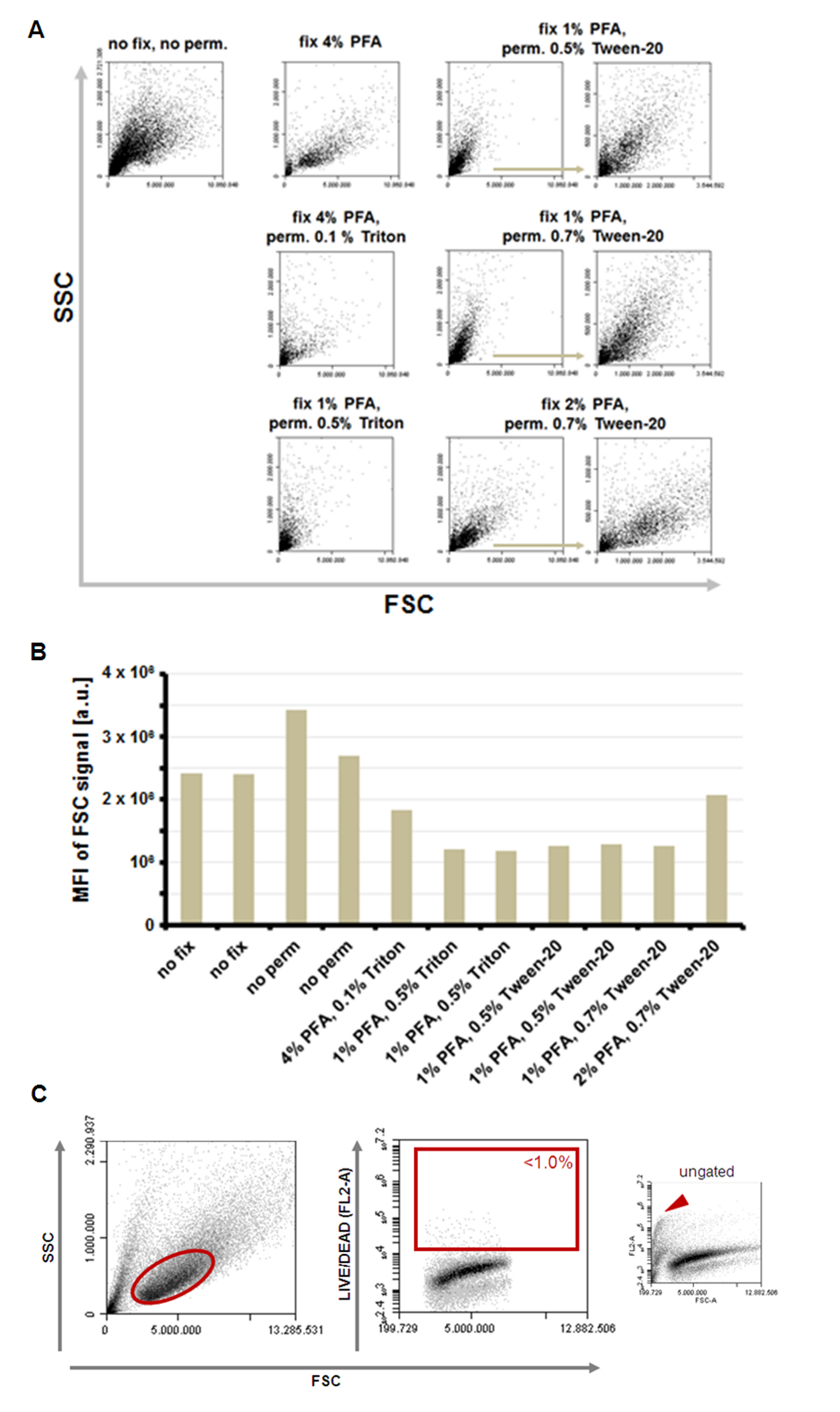

Supplement: Figure S2 — (A) Incubation with fixative and permeabilization buffers as indicated alters flow cytometric forward and side scatter properties (SH-SY5Y cell line). Adjusting FSC resolution (arrows) enables proper visual representation and subsequent analysis of the overall population (far right column of panels). (B) Forward scatter signal is particularly affected by permeabilization with either detergent. Mean fluorescence intensity (MFI) of a single representative experiment is shown. (C) Viability assessment within FSC/SSC-based gate using a fixable red-fluorescent live/dead labeling dye. (TIF) [file pone.0068519.s002.tif]

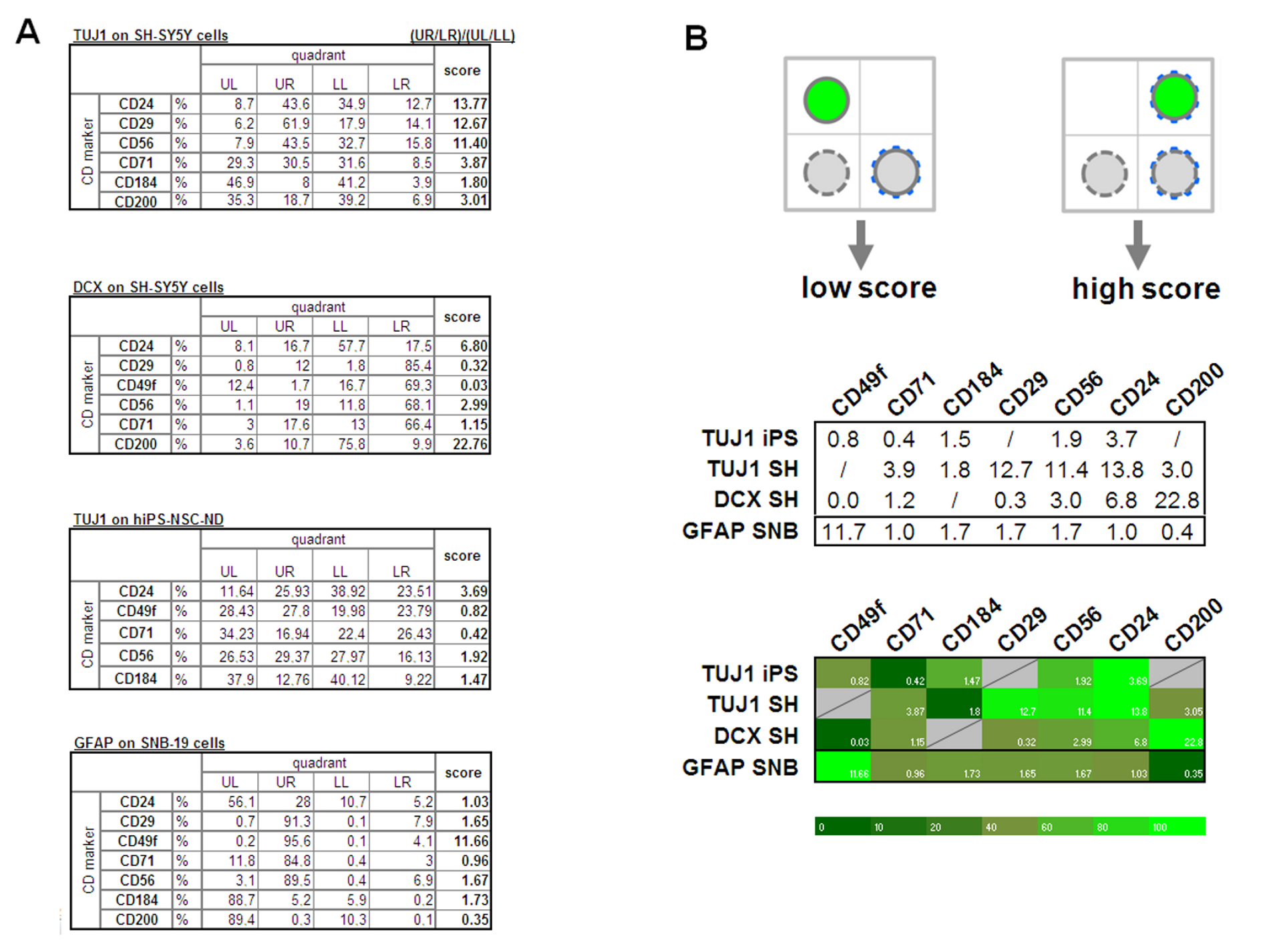

Supplement: Figure S3 — (A) Co-expression scores were calculated by determining the ratio of cells (percentage) present in upper right (UR) to lower right (LR) quadrants over the ratio of upper left (UL) to lower left (LL) quadrants, where surface antigen staining was shown on the abscissa and intracellular stain on the ordinate of the respective flow plots (as applied throughout this manuscript) [Coexpression score= (UR/LR)/(UL/LL)]. A percentage value of 0.1% was assigned where no cells were present in a quadrant (see SNB-19, CD200 stain). Surface antigens co-stained with DCX were quantified on SH-SY5Y cells. Surface antigens co-stained with TUJ1 were quantified on SH-SY5Y cells and neuronally differentiating cultures derived from human iPS cells, and surface antigens co-stained with GFAP were quantified on SNB-19 cells. (B) Using the conditional formatting function in Microsoft Excel, a dark to light-green color scale was applied to each one of the intracellular co-stained data sets to generate co-expression heatmap (see Figure 6D ). Note differential clustering of scores for SNB-19 glial cells versus the other cell sources capable of neuronal differentiation. (TIF) [file pone.0068519.s003.tif]
